# Determinants of adolescent pregnancy in East Africa: a systematic review and meta-analysis

**DOI:** 10.11604/pamj.2025.52.42.48583

**Published:** 2025-09-29

**Authors:** Sandra Isano, Theogene Uwizeyimana, Karl Blanchet

**Affiliations:** 1 Geneva Centre of Humanitarian Studies, University of Geneva, Switzerland, City of Geneva,; 2 University of Global Health Equity, Burera, Rwanda

**Keywords:** Adolescent pregnancy, East Africa, socio-economic determinants

## Abstract

Adolescent pregnancy remains a major public health and socio-economic challenge in East Africa, where rates remain disproportionately high compared to global trends. This systematic review and meta-analysis aimed to estimate the pooled prevalence of adolescent pregnancy and examine its association with key socio-economic factors across East African countries. Following PRISMA 2020 and Joanna Briggs Institute (JBI) guidelines, a comprehensive search of five electronic databases and national demographic surveys was conducted to identify relevant studies published between January 2013 and December 2023. Nineteen studies were included, covering cross-sectional, cohort, and case-control designs across Uganda, Kenya, Tanzania, Rwanda, Burundi, Democratic Republic of Congo, and South Sudan. The pooled prevalence of adolescent pregnancy was 23.6% (95% CI: 15.0-33.4), with the lowest rate observed in Rwanda (5.2%) and the highest in Tanzania (34.0%). Socio-economic determinants showed strong associations with adolescent pregnancy. Adolescents with employment were significantly less likely to become pregnant (pooled OR = 0.27; 95% CI: 0.03-0.50), while low educational attainment and poverty were also associated with increased pregnancy risk, although pooled effects were not statistically significant. The findings underscore the multifaceted nature of adolescent pregnancy in East Africa, driven by structural inequalities, limited access to education and reproductive health services, cultural norms, and economic vulnerability. Addressing this issue requires multisectoral strategies that promote girls' education, improve youth employment opportunities, expand access to adolescent-friendly sexual and reproductive health services, and challenge harmful social norms. Context-specific interventions that reflect national and subnational realities are essential. Future research should incorporate longitudinal and qualitative approaches to capture the lived experiences of adolescents and the long-term consequences of early pregnancy. Centering adolescent voices and investing in holistic, rights-based policies will be critical to reducing adolescent pregnancy and advancing gender equity across the region.

## Introduction

Adolescent pregnancy remains a critical global health issue, especially in low-income settings where its prevalence is alarmingly high [1]. Each year, approximately 21 million girls aged 15 to 19 in low-income countries become pregnant. Notably, nearly half of these pregnancies (49%) are unintended, with about 16 million resulting in childbirth and approximately 3.2 million ending in abortion [2,3]. Although there has been a global decline in adolescent birth rates, sub-Saharan Africa continues to face severe challenges, with over 100 births per 1,000 women in this age group [4]. This issue extends beyond individual health, representing a complex public health crisis with significant implications for socio-economic development and gender equality [5].

In some cultures, pregnancy is viewed positively as a blessing [6,7]. However, for adolescent girls, early pregnancy often results in severe and detrimental consequences. One of the most significant impacts is the disruption of education; many young mothers are compelled to leave school, which severely limits their future economic opportunities and perpetuates cycles of poverty. This interruption not only affects their immediate financial stability but also hinders their long-term career prospects [8]. Furthermore, early pregnancy introduces additional challenges, such as increased risk of unemployment. Research highlights that adolescent pregnancy often leads to joblessness due to both a lack of educational qualifications and the stigma associated with early motherhood [9].This combination of factors further restricts their ability to achieve economic independence and secure stable employment [10].

The adverse outcomes of adolescent pregnancy extend beyond the individual, significantly impacting the health and development of the child. Children born to adolescent mothers are at a higher risk for low birth weight, premature birth, and developmental delays [10]. These children often face additional challenges due to the mother's immaturity and potential instability in the mother-child relationship [11,12]. In some cases, these children are raised by grandparents or other relatives, which can result in frequent changes in caregivers [13]. This instability can increase the risk of neglect and negatively affect the child's overall development [13,14].

Adolescent pregnancy is a significant concern with varying social determinants across different regions of the world. Among the factors contributing to adolescent pregnancy are poverty, cultural and religious beliefs, early marriages, and limited access to education, among others. These factors interact in complex ways, but their impact can differ greatly depending on the socio-cultural and economic context of each region [9,11,15]. For example, in many parts of sub-Saharan Africa, traditional practices and religious beliefs significantly contribute to high rates of adolescent pregnancy. Early marriages and cultural norms are key factors influencing these rates [16,17]. In other regions, economic instability and limited access to comprehensive sexual education may be more prominent [18]. In Eastern Africa, the determinants of adolescent pregnancy are shaped by a unique combination of cultural practices, educational barriers, and economic conditions. Traditional beliefs and early marriage customs are prevalent, while access to education varies significantly. Poverty exacerbates these issues by restricting access to healthcare and sexual education [2,19,20].

Existing literature has identified the factors contributing to adolescent pregnancy in sub-Saharan Africa, highlighting its high prevalence. While recent reviews stress the need for targeted interventions, they often provide broad, generalized insights that may not adequately reflect the specific context of individual regions [3,4,21,22]. A systematic review focused specifically on East Africa is necessary to fill this gap. By examining the unique socio-cultural, economic, and environmental factors influencing adolescent pregnancy in East Africa, this review aims to generate a more nuanced understanding. Such insights are crucial for developing tailored interventions that more effectively address the challenges unique to adolescents in this region.

## Methods

**Study design and search strategy:** a systematic review and meta-analysis were conducted to estimate the pooled prevalence of adolescent pregnancy and assess associated socio-economic factors in East Africa. The review followed PRISMA 2020 guidelines and adhered to the Joanna Briggs Institute (JBI) methodology for systematic reviews [23-25]. A comprehensive search strategy was used to identify both published and unpublished literature. Records were retrieved from MEDLINE (n = 140), R Discovery (n = 62), Scite AI (n = 540), Elicit AI (n = 64), and Demographic and Health Survey (DHS) reports (n = 5), yielding a total of 811 records. Boolean operators (AND, OR) were used to combine search terms including “adolescent pregnancy,” “teenage pregnancy,” “East Africa,” and “risk factors.” Reference lists of included studies were screened to identify additional eligible sources.

**Study selection and eligibility criteria:** following deduplication, 277 records were removed, and 180 were excluded by automated filters. After screening 140 titles and abstracts, 113 records were excluded. Of 27 full-text articles assessed, 8 were excluded due to ineligible study designs or exposures. Nineteen studies were included in the final analysis.

***Inclusion criteria:*** published in English (2013-2023); focused on adolescent girls aged 13-19 years in East Africa; reported prevalence or socio-economic risk factors for adolescent pregnancy and used observational study designs (cross-sectional, cohort, case-control) or DHS data

***Exclusion criteria:*** focused on male adolescents or non-empirical studies; review articles or qualitative research and ineligible design or exposure focus.

**Definition of adolescent pregnancy:** adolescent pregnancy was defined as pregnancy occurring among girls aged 13-19 years. Studies reporting first births, current pregnancies, or proxy indicators from DHS reports were included.

**Quality assessment and data extraction:** the JBI critical appraisal checklist was used to assess methodological quality. Two reviewers independently extracted data using JBI SUMARI. Extracted data included author, year, study location, design, sample size, target population, outcomes, and key findings. Discrepancies were resolved by consensus.

**Heterogeneity and publication bias:** statistical heterogeneity was assessed using the I^2^ statistic. Thresholds of 25%, 50%, and 75% were used to denote low, moderate, and high heterogeneity [26]. Egger's regression test was used to evaluate publication bias [27]. Where bias was detected (p < 0.05), the Duval and Tweedie trim-and-fill method was applied [28].

**Data analysis and presentation:** data were entered into Microsoft Excel, and meta-analysis was conducted using the Metafor package in R [29]. A random-effects model was used to account for heterogeneity across studies. Forest plot was used to present the pooled prevalence of adolescent pregnancy, while summary tables were used to report associations with key socio-economic factors such as employment status, education, and poverty, expressed as odds ratios (ORs) with corresponding 95% confidence intervals (CIs).

## Results

**Figure 1:**
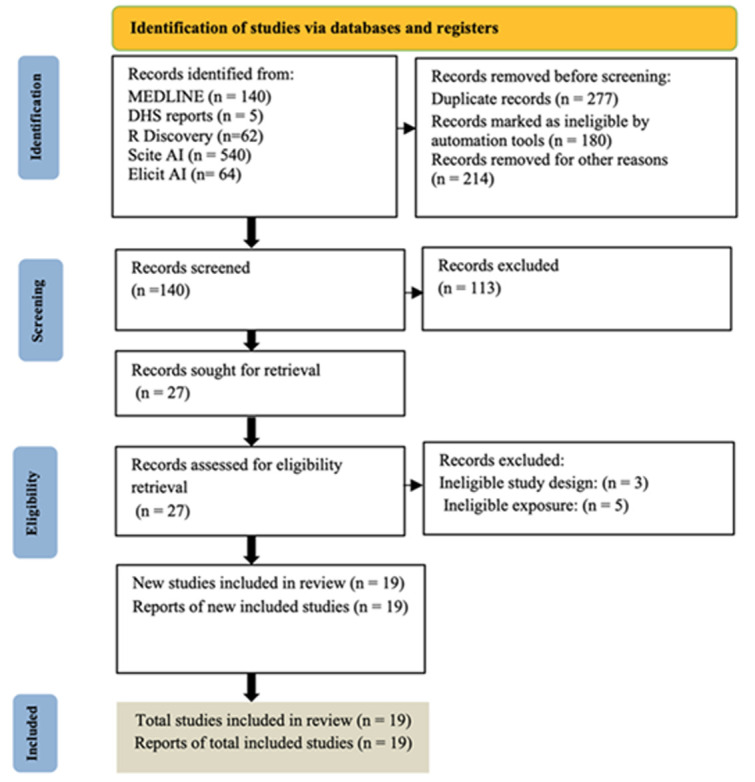
search results, study selection, and inclusion process

**Study selection:** out of 811 records identified, 19 studies met inclusion criteria and were included in the meta-analysis. The study selection process is outlined in Figure 1.

**Characteristics of included studies:** the 19 included studies were conducted between 2013 and 2023 across East African countries, including Uganda, Kenya, Tanzania, Rwanda, Burundi, Democratic Republic of Congo, and South Sudan. The majority were cross-sectional (n = 12), with cohort (n = 5) and case-control (n = 2) designs also represented. Adolescents aged 13-19 years were the primary population of interest. Key outcomes included the prevalence of adolescent pregnancy and associations with socio-economic variables. Full study details are provided in Table 1.

**Table 1:** characteristics of included sources of evidence

Authors	Study Design	Country	Population	Key outcome measured
Amongin D *et al*. (2020)	Cross-sectional	Uganda	Women who had repeat adolescent births	Repeat adolescent births and factors associated with them
Burke HM *et al*. (2018)	Cross-sectional	Uganda	Adolescents 15-19 years	Correlates of rapid repeat pregnancy
Byonanebye J *et al*. (2020)	Cross-sectional	Uganda	Adolescents 15-19 years	Geographic variation, risk factors for teenage pregnancy
Chemutai V *et al*. (2022)	Cross-Sectional	Uganda	Adolescents 15-19 years	Prevalence and associated factors of teenage pregnancy
Mbabazi C *et al*. (2021)	Cross-sectional	Uganda	Adolescents 15-19 years	Factors associated with adolescent pregnancy
Moshi FV *et al*. (2023)	Cross-sectional	Tanzania	Adolescents 15-19 years	Magnitude and associated factors of teenage pregnancy
Musinguzi M *et al*. (2022)	Cross-Sectional	Uganda	Adolescents 15-19 years	Prevalence and correlates of teenage pregnancy during COVID-19
Mutea L *et al*. (2022)	Cross-sectional	Kenya	Adolescents 15-19 years	Trends and determinants of adolescent pregnancy
Ngoda OA *et al*. (2023)	Cross-sectional	Tanzania	Adolescents 15-19 years	Trends and factors associated with adolescent pregnancies
Ochen AM *et al*. (2019)	Case-control	Uganda	Adolescents 13-19 years	Predictors of teenage pregnancy
Okigbo CC *et al*. (2015)	Cross-sectional	Kenya	Adolescents 15-24 years	Sexual activity and pregnancy among unmarried young women
Okot C *et al*. (2023)	Cross-sectional	Uganda	Adolescents 13-17 years	Prevalence and associated factors of teenage pregnancy
Swahn MH *et al*. (2022)	Cross-sectional	Uganda	Adolescents 12-18 years	Demographic and psychosocial risk factors for adolescent pregnancies
Burundi DHS 2017	DHS	Burundi	Adolescents 15-19 years	Prevalence of adolescent pregnancy
Kenya DHS 2022	DHS	Kenya	Adolescents 15-19 years	Prevalence of adolescent pregnancy
Rwanda DHS 2019-20	DHS	Rwanda	Adolescents 15-19 years	Prevalence of adolescent pregnancy
Tanzania DHS 2022	DHS	Tanzania	Adolescents 15-19 years	Prevalence of adolescent pregnancy
Uganda DHS 2022	DHS	Uganda	Adolescents 15-19 years	Prevalence of adolescent pregnancy
Democratic Republic of Congo DHS 2023-2024	DHS	DRC	Adolescents 15-19 years	Prevalence of adolescent pregnancy

**Figure 2:**
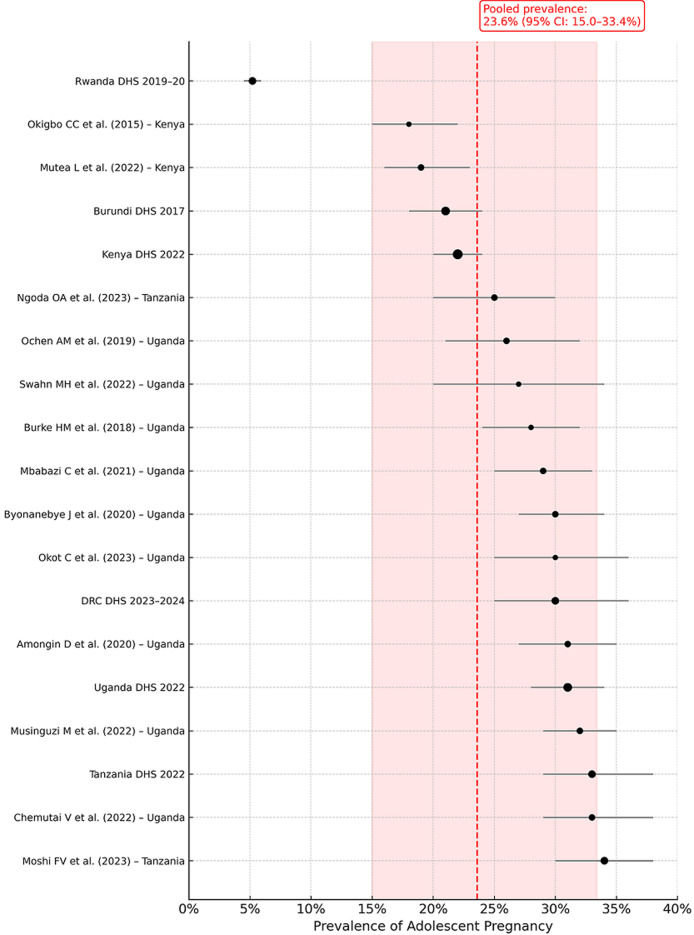
pooled prevalence of adolescent pregnancy in East Africa

**Prevalence of adolescent pregnancy in East Africa:** the prevalence of adolescent pregnancy varied from 5.2% to 34.0% across studies. The lowest prevalence was reported in Rwanda (Rwanda DHS 2019-20: 5.2%, 95% CI: 4.5-5.9), and the highest in Tanzania (Moshi *et al*. 2023: 34.0%, 95% CI: 30.0-38.0). The overall pooled prevalence across all 19 studies was 23.6% (95% CI: 15.0-33.4), suggesting that nearly one in four adolescent girls in East Africa has experienced pregnancy. There was substantial heterogeneity among studies (I^2^ = 99.8%), likely reflecting variation in geography, methodology, and populations. Figure 2 presents the pooled prevalence forest plot.

### Socio-economic factors associated with adolescent pregnancy

***Employment status*:** four studies examined the relationship between employment and adolescent pregnancy. One Rwandan study reported a significant positive association (OR = 1.51; 95% CI: 1.03-2.64), while the remaining three showed non-significant findings. The pooled OR from the random-effects model indicated that employed adolescents had significantly lower odds of pregnancy (OR = 0.27; 95% CI: 0.03-0.50). No heterogeneity was detected (I^2^ = 0%, p = 0.28) Table 2.

**Table 2:** association between employment status and adolescent pregnancy

Study	Country	Odds Ratio [95% CI]	Significant	Weight (%)
Chemutai V *et al*. (2022)	Uganda	0.27 [95% CI: 0.08–0.96]	Yes	7.6%
Okigbo CC *et al*. (2015)	Kenya	1.51 [95% CI: 0.35–6.49]	No	4.6%
Amongin D *et al*. (2020)	Uganda	0.80 [95% CI: 0.40–1.60]	No	76.0%
Rwanda DHS 2019–20	Rwanda	1.51 [95% CI: 1.03–2.64]	Yes	11.8%

Pooled estimate: OR = 0.27 [95% CI: 0.03–0.50]; I^2^ = 0%, p = 0.28

***Education level:*** six studies assessed education. One study in 2015 showed a protective effect (OR = 0.20; 95% CI: 0.09-0.45), while four others found significant associations linking lower education with higher pregnancy risk. One study showed a non-significant effect. The pooled estimate was not statistically significant (OR = 1.06; 95% CI: 0.15-1.97), with substantial heterogeneity (I^2^ = 92.02%). See Table 3.

**Table 3:** association between education and adolescent pregnancy

Study	Country	Odds Ratio [95% CI]	Significant	Weight (%)
Chemutai V *et al*. (2022)	Uganda	9.58 [95% CI: 3.49-26.31]	Yes	5.1%
Okigbo CC *et al*. (2015)	Kenya	0.20 [95% CI: 0.09-0.45]	Yes	8.0%
Swahn MH *et al*. (2022)	Uganda	1.22 [95% CI: 0.64-2.32]	No	12.9%
Rwanda DHS 2019-20	Rwanda	5.87 [95% CI: 2.44-14.30]	Yes	6.6%
Okot C *et al*. (2023)	Uganda	10.18 [95% CI: 1.45-71.52]	Yes	1.4%
Amongin D *et al*. (2020)	Uganda	2.51 [95% CI: 1.09-5.70]	Yes	7.7%

Pooled estimate: OR = 1.06 [95% CI: 0.15-1.97]; I^2^ = 92.02%

**Poverty:** four studies evaluated poverty. One Kenyan study reported a protective effect (OR = 0.30; 95% CI: 0.10-0.90), while two Ugandan studies found significant positive associations (Chemutai *et al*., OR = 2.80; 95% CI: 1.09-7.17; Amongin *et al*. OR = 1.70; 95% CI: 1.25-2.32). One Rwandan study showed a non-significant result. The pooled analysis found no significant overall effect (OR = 0.11; 95% CI: âˆ’0.75 to 0.96), with moderate heterogeneity (I^2^ = 82.8%, p = 0.005) Table 4.

**Table 4:** association between poverty and adolescent pregnancy

Study	Country	Odds Ratio [95% CI]	Significant	Weight (%)
Chemutai V *et al*. (2022)	Uganda	2.80 [95% CI: 1.09–7.17]	Yes	7.8%
Okigbo CC *et al*. (2015)	Kenya	0.30 [95% CI: 0.10–0.90]	Yes	5.7%
Rwanda DHS 2019–20	Rwanda	0.85 [95% CI: 0.41–1.75]	No	13.3%
Amongin D *et al*. (2020)	Uganda	1.70 [95% CI: 1.25–2.32]	Yes	73.2%

Pooled estimate: OR = 0.11 [95% CI: –0.75–0.96]; I^2^ = 82.8%, p = 0.005

## Discussion

The findings from this systematic review and meta-analysis provide an overview of the prevalence and socio-economic determinants of adolescent pregnancy in East Africa. The pooled prevalence of adolescent pregnancy ranged from 5.2% to 34.0%, with the highest rate observed in Tanzania and the lowest in Rwanda. These findings align with previous research, which has reported similarly high rates of adolescent pregnancy across sub-Saharan Africa. In some parts of the region, including Tanzania, Uganda, and Kenya, adolescent birth rates exceed 100 births per 1,000 women, highlighting the magnitude of the issue [16,18,19]. The relatively lower prevalence observed in Rwanda suggests that national policies and interventions may be having a measurable impact, though the overall burden across East Africa remains substantial [30].

Importantly, our findings underscore the significant role of socio-economic factors—including poverty, low educational attainment, and unemployment—in influencing adolescent pregnancy. Adolescents from poor households were found to be at a higher risk of early pregnancy. This is largely attributed to limited access to healthcare services, including contraception and sexual and reproductive health information, as well as the economic pressures that may lead to transactional sex or early marriage [18,31]. These dynamics are well documented in the broader literature, which consistently highlights how poverty exacerbates vulnerability by restricting adolescents´ access to protective resources and support systems [17-19].

Education also emerged as a key determinant of adolescent pregnancy. Our meta-analysis found that while the overall association between education and adolescent pregnancy was not statistically significant, individual studies revealed strong links between low educational attainment and increased risk of early pregnancy. This is consistent with prior research demonstrating that girls who stay in school, particularly through secondary education, are significantly less likely to become pregnant [32]. Schools not only serve as platforms for delivering reproductive health education but also help delay marriage and childbearing and empower girls to make informed decisions about their bodies and futures. Adolescents who drop out of school are more vulnerable to early pregnancy, which can further limit their access to employment and perpetuate cycles of poverty [18].

Unemployment, another important factor in our analysis, also showed a significant relationship with adolescent pregnancy. While only one of the included studies found a statistically significant association, the pooled effect indicated that unemployment increased the likelihood of adolescent pregnancy. This is supported by studies suggesting that limited economic opportunities for young women increase their vulnerability to early childbearing, particularly in contexts where early motherhood is socially normalised or economically incentivised [21]. In the absence of meaningful employment, adolescent girls may view motherhood as their only viable life path, reinforcing gender-based economic disparities and limiting their potential.

Moreover, poverty was revealed as a foundational factor influencing all other socio-economic variables. While our findings showed mixed associations between poverty and adolescent pregnancy—with some studies showing significant positive effects and others showing no effect—the broader literature identifies poverty as a root cause that shapes adolescent girls´ reproductive trajectories [16,33]. Poor adolescents are less likely to remain in school, access reproductive health services, or resist social pressures that encourage early sex and marriage. Interventions addressing adolescent pregnancy must therefore go beyond individual behaviour change and incorporate structural approaches, such as conditional cash transfers, social protection programs, and economic empowerment initiatives.

As highlighted in our findings, the evidence synthesised in this review is based on data extracted from the included primary studies summarised in Table 1 [34-50]. These studies provide the empirical foundation for the prevalence estimates and socio-economic determinants discussed above. By integrating data across these diverse sources, this review offers a comprehensive understanding of adolescent pregnancy in East Africa and underscores the need for context-specific interventions informed by local evidence.

## Conclusion

In summary, our review reinforces the complex interplay between education, employment, and poverty in shaping adolescent pregnancy outcomes in East Africa. While individual interventions can help, sustainable reductions in adolescent pregnancy require a multisectoral approach that addresses the broader social determinants of health. Future policy should prioritise expanding access to education, improving youth employment opportunities, and implementing poverty alleviation strategies, particularly for adolescent girls. Such an integrated strategy is crucial not only to reduce adolescent pregnancy but also to improve long-term health, educational, and economic outcomes for young women in the region.

### What is known about this topic

- Adolescent pregnancy remains a major public health issue in sub-Saharan Africa, with significant health and socio-economic consequences;
- Factors such as low educational attainment, poverty, and early marriage are commonly associated with increased rates of adolescent pregnancy;
- Despite multiple interventions, progress in reducing adolescent pregnancy in East Africa has been uneven and slow.

### What this study adds

- This systematic review and meta-analysis provides pooled evidence on the strength and consistency of associations between key risk factors and adolescent pregnancy in East Africa;
- The findings highlight significant heterogeneity across studies and show that while some factors (e.g., low education) consistently show strong associations, others (e.g., poverty, unemployment) present mixed evidence;
- The study underscores the need for context-specific, multisectoral interventions that address underlying structural determinants such as poverty, education, and access to adolescent-friendly reproductive health services.
